# Use of Multiple Machine Learning Approaches for Selecting Urothelial Cancer-Specific DNA Methylation Biomarkers in Urine

**DOI:** 10.3390/ijms25020738

**Published:** 2024-01-06

**Authors:** Christina U. Köhler, Karin Schork, Michael Turewicz, Martin Eisenacher, Florian Roghmann, Joachim Noldus, Katrin Marcus, Thomas Brüning, Heiko U. Käfferlein

**Affiliations:** 1 Institute for Prevention and Occupational Medicine of the German Social Accident Insurance, Ruhr University Bochum (IPA), Bürkle-de-la-Camp Platz 1, 44789 Bochum, Germany; christina.koehler@dguv.de (C.U.K.);; 2 Medizinisches Proteom-Center, Medical Faculty, Ruhr-University Bochum and Medical Proteome Analysis, Center for Protein Diagnostics (PRODI), Gesundheitscampus 4, 44081 Bochum, Germany; 3 Department of Urology, Marien Hospital Herne, University Hospital of the Ruhr University Bochum, Hölkeskampring 40, 44625 Herne, Germany

**Keywords:** urothelial cancer, DNA methylation biomarker, random forest, boosted trees, LASSO

## Abstract

Diagnosing urothelial cancer (UCa) via invasive cystoscopy is painful, specifically in men, and can cause infection and bleeding. Because the UCa risk is higher for male patients, urinary non-invasive UCa biomarkers are highly desired to stratify men for invasive cystoscopy. We previously identified multiple DNA methylation sites in urine samples that detect UCa with a high sensitivity and specificity in men. Here, we identified the most relevant markers by employing multiple statistical approaches and machine learning (random forest, boosted trees, LASSO) using a dataset of 251 male UCa patients and 111 controls. Three CpG sites located in *ALOX5*, *TRPS1* and an intergenic region on chromosome 16 have been concordantly selected by all approaches, and their combination in a single decision matrix for clinical use was tested based on their respective thresholds of the individual CpGs. The combination of *ALOX5* and *TRPS1* yielded the best overall sensitivity (61%) at a pre-set specificity of 95%. This combination exceeded both the diagnostic performance of the most sensitive bioinformatic approach and that of the best single CpG. In summary, we showed that overlap analysis of multiple statistical approaches identifies the most reliable biomarkers for UCa in a male collective. The results may assist in stratifying men for cystoscopy.

## 1. Introduction

Urothelial carcinoma (UCa) is among the most frequent cancers of the world and more likely to occur in men [1]. The standard for diagnosing primary and recurrent UCa is urinary cystoscopy, which is an invasive and uncomfortable procedure [2]. The cytological evaluation of the urinary sediment has been widely used as a non-invasive method to precede cystoscopy and is highly specific [3]. However, it has a limited sensitivity for low-grade tumors [3,4]. Though invasive cystoscopy has no alternative in patients presenting with macrohematuria in a doctor’s office, the decision as to whether perform cystoscopy in the case of microhematuria is much more challenging. In such cases, a biomarker-based approach might aid decision making rather than only relying on common urothelial cancer-associated risk factors such as smoking status, age and male gender. Therefore, non-invasive biomarkers for UCa in urine are obviously needed, specifically in men.

Due to the direct contact of urine and urothelium, urothelial cancer cells or cancer cell components are released into the urine. Numerous approaches have been carried out to identify UCa by the use of urinary molecular biomarkers [5,6,7,8,9,10,11]. However, none of the used approaches or identified biomarkers have been included in a clinical routine or even in guidelines, possibly due to the complex nature of molecular signatures that, in part, require a specialized readout software [10,11].

We have previously identified a set of urinary DNA methylation biomarkers that detect UCa with a high sensitivity and specificity specifically in male patients and independent of grade, stage and hematuria. Upon the initial screening in urinary sediment, DNA methylation was verified by using an independent method and it was confirmed in matched tissue specimens [12]. The analyses included multiple candidate CpG sites within 10 amplicons and, based on ROC analyses, we suggested the use of the most relevant single CpGs to diagnose UCa.

In recent years, machine learning has become increasingly commonly employed for analyzing DNA methylation data to diagnose diseases [13,14,15,16,17,18] and disease subtypes [16,19,20,21] or to discriminate tissue and tumor origins [22,23,24,25,26]. Herein, random forest has been primarily used. However, boosted tree approaches have also gained attention [23]. In addition, combinations of machine learning strategies were discussed to improve data outcomes [17,22].

Here, we applied different modeling and machine learning approaches including random forest (RF), boosted trees (BT) and LASSO to our previously identified targets to further improve parameter selection strategies. In addition, we evaluated the performance of each single CpG via ROC analyses. Assuming that CpG sites selected by several different statistical and machine learning approaches are exceptionally meaningful, our aim was to combine these concordantly selected sites in a simple diagnostic decision matrix based on their respective individual ROC cutoffs. The latter should be easily employable for clinicians without the use of bioinformatics.

## 2. Results and Discussion

Genome-wide screening technologies enable the detection of disease-specific signatures. However, they often yield numerous potential biomarkers and subsequently require a careful selection of the diagnostically most meaningful sites. In our previous study, we suggested the use of single CpGs for UCa detection [12]. Meanwhile, machine learning algorithms have been used successfully to create disease classifiers. The most widely described machine learning process with respect to DNA methylation data has been random forest (RF), favoring either accuracy or specificity [14,15,16,17,18,20,21,23]. Furthermore, boosted trees (BT), promoting either accuracy or specificity, have also been successfully applied [16,23]. In addition, LASSO has been used for DNA methylation analyses before [17,18,20].

Most of the methods have been used as “stand-alone“ methods to identify disease classifiers. Only a few studies have reported the use of more than just one statistical or bioinformatical approach for methylation biomarker identification. One strategy has been to combine several methods in a series. For example, targets are first selected via LASSO or principal component analysis (PCA) and then subject to RF or support vector machines (SVM) for the development of a final classifier [17,22]. Alternatively, RF and LASSO were performed in parallel and the overlapping CpGs were combined to obtain a final classifier via logistic regression analysis [18].

In the present study, we applied three important methods (RF, BT, LASSO) in parallel on a large dataset of UCa patients and controls. To minimize method-inherent disadvantages or biases of the respective singular approaches, we determined the overlap of those sites that were identified by the different tests applied. Instead of combining the selected sites in a complex classifier or model, we analyzed all possible combinations of the selected biomarker sites in “or-matrices” based on the thresholds in the individual ROC analyses and at a pre-set 95% specificity to minimize false positive results and then we compared the diagnostic value of these combinations to the sensitivity and specificity of the singular markers picked. According to this comparison, we developed a simple decision matrix for easy application in clinical practice to select individuals for invasive cystoscopy. Similar “simple“ matrices have been used for DNA methylation analyses in earlier biomarker studies [27,28,29].

When comparing the results of the various parameter selection methods, the RF approach selected about a four-fold higher number of sites (49 and 43 CpGs in RF when optimized for either accuracy or specificity) compared to the BT approach (10 and 13 CpGs, respectively) (Table 1, Tables S4–S7, and Figures S2–S5). LASSO was the most restrictive and only selected four sites (Table 1). This finding is in accordance with a study that compared RF and LASSO for identifying classifiers of gastric cancer and where the number of parameters selected by RF was much higher than of those picked by LASSO [18].

A ranking of important diagnostic parameters revealed very similar AUCs for the different methods. AUC values of single sites (e.g., approx. 86% for CpG unit 78_2.3) were marginally higher than those of RF_spec_, RF_acc,_ BT_acc_ and LASSO (approx. 85%) and slightly higher than those of BT_spec_ (84%, Table 1). In contrast, the sensitivities between the various approaches (at a pre-defined specificity of 95%) displayed more pronounced differences. Single CpGs displayed a maximum sensitivity of approx. 58% (for CpG 2_6) and thus outperformed BT_acc_ (approx. 53%). The sensitivities of LASSO and RF_spec_ (both approx. 52%) were only slightly higher than those obtained in RF_acc_ (51%). BT_spec_ ranked least with a sensitivity of approx. 50% at 95% specificity (Table 1).

Among the 65 sites analyzed, the majority of CpGs (42) analyzed by RF analyses were concomitantly selected by both RF_acc_ (Table 1, Tables S4 and S8) and RF_spec_ (Table 1, Tables S5 and S8), whereas regarding BT analyses only seven CpGs were concomitantly selected by both BT_acc_ (Table 1, Tables S6 and S8) and BT_spec_ (Table 1, Tables S7 and S8). All seven potential classifiers identified by the BT approach were also selected by the RF approach (Table 1 and Table S8). Among the four sites identified by LASSO (Table 1), three sites (02_CpG_6, 35_CpG_7 and 78_CpG_2.3) were also picked by all RF and BT approaches (Table S8). Therefore, these three sites (*ALOX5*, *TRPS1* and the intergenic region on chromosome 16) were judged the most relevant biomarkers to be used for UCa diagnosis and, consequently, were considered for the development of the decision matrix introduced in the present study (Table 2 and Table S8). For these three candidates—alone or in any combination—the sensitivities and specificities were determined based on the thresholds of the respective single sites at 95% specificity (Table 2 and Table S3). Both diagnostic parameters were calculated for the overall collective, including patients with primary tumors and those that had suffered from UCa before (Table 2). In addition, we analyzed the sensitivities and specificities stratified for a history of UCa (Table 2).

For a good diagnostic tool, a high specificity is required to prevent unnecessary cystoscopies. Therefore, we only selected sites or panels with a specificity of at least 95% for further evaluation in the overall collective. Among the resulting options, *ALOX5* (coding for arachidonate 5-lipoxygenase) had the best specificity (96%) and a reasonable sensitivity (58%). Stratified by history of UCa, the specificity was even better with 98% at a good sensitivity of 70% in patients with primary UCa. However, both specificity (94%) and sensitivity (40%) were considerably reduced in patients with UCa history (Table 2). A simple “or” matrix applying a combination of *ALOX5* and *TRPS1* (coding for the transcriptional repressor GATA binding 1) increased the sensitivity in the overall collective by 3% at only a 1% drop in specificity when compared to *ALOX5* alone and was therefore considered the best option for UCa detection according to our selection criteria described below (see Section 3). In patients with no former UCa, the sensitivity was also increased by 2% at an identically high specificity (98%, Table 2). However, in patients with UCa history, the discussed combination yielded only 43% sensitivity. For individuals with previous UCa, this was the best value among the selected sites and their combinations and was increased by 3% when compared to the best single CpG; however, the specificity was reduced by 4% when compared to *ALOX5* alone (Table 2).

As already speculated by other authors, a reduced sensitivity in patients experiencing recurrences probably results from the fact that patients with UCa history are under surveillance. Thus, tumors are still small and release only a few cells into the urine that might be detected by molecular approaches, although, admittedly, cancerous lesions may already become visible when employing cystoscopy [30,31]. However, small non-invasive lesions that do not cause pain or discomfort usually have a small probability to progress and should become apparent in a later surveillance visit.

Generally, increased sensitivities (most likely accompanied by decreased specificities) are observed when using “or” combinations for biomarkers, i.e., when two or more biomarkers must exceed a threshold for the specimen to be positive. In addition, it should be kept in mind that, compared to other solid cancers, UCa is exceptional due to its high recurrence rates. Therefore, the need for invasive cystoscopy is different in patients with primary UCa compared to those under surveillance for recurrent UCa: patients with a history of UCa became used to regular follow-up investigations and they expect invasive interventions compared to patients that are under UCa suspicion for the first time. Therefore, a lower specificity in terms of an increased number of false-positive results is acceptable in patients with recurrent UCa, specifically in patients that previously suffered from non-muscle invasive bladder cancer with only low and intermediate risk. In addition, surveillance patients might experience a negative cystoscopy result (obtained upon a preceding false-positive biomarker test result) as a relief that outweighs the inconveniences during cystoscopy. Therefore, we advocate for the more sensitive (but less specific) “or” combination of *ALOX5* and *TRPS1* for guiding cystoscopies in patients under surveillance for recurrent UCa. 

In patients without a history of UCa, the marker combination “*ALOX5* or *TRPS1*” is both more sensitive (72% vs. 43%) and highly specific (98% vs. 90%, Table 2) when compared to people with UCa history. We therefore also recommend the marker combination of *ALOX5* and *TRPS1* for patients that are under suspicion of UCa for the first time. The latter might not return for necessary future investigations after experiencing a painful and unnecessary investigation via invasive cystoscopy; therefore, sparing patients from unnecessary cystoscopies (enabled by a high biomarker specificity) is even more important in primary UCa patients when compared to those under surveillance of recurrent UCa. 

To our knowledge, neither *TRPS1* nor *ALOX5* was previously identified as a differentially methylated target in UCa except from our own group [12]. However, there are studies showing a significantly higher expression of ALOX 5 at the levels of both protein and RNA expression in UCa than in cancer-free urothelium [32,33,34]. Furthermore, ALOX5 inhibitors exhibited growth suppression in UCa cell lines expressing ALOX5. Inhibitor-mediated growth inhibition could be diminished by 5-HETE as a product of ALOX5-dependent metabolism, suggesting that ALOX-5-dependent metabolism of arachidonic acid—predominantly via 5-HETE production—is involved in the biology of UCa [35]. As far as TRPS1 is concerned, we only found articles reporting no expression at the protein level in UCa; however, expression levels in non-cancerous urothelial tissue were not reported for comparison [36]. 

Our approach here is unique because, even for a single biomarker, we pre-set the specificity to a high level (95%). In contrast, previous studies mostly balanced sensitivity and specificity when reporting their results and the collectives were differently composed regarding grading, staging and tumor history. Therefore, the diagnostic performances in terms of specificity and sensitivity reported here are generally hard to compare to those previously reported by other authors. Few reviews selecting markers and panels with high diagnostic precision summarized sensitivities and specificities and reported studies yielding sensitivities > 60% at 95% specificity [6,7,10,11,13,37,38]. However, none of these studies investigated as many cases and/or controls as we investigated; thus, it is not clear whether the same sensitivities would have been reported in larger collectives. 

In our overall collective, the sensitivity of each of our single or combined markers exceeded the overall value obtained for urine cytology in a large meta-analysis (around 30%, [3]) at a similar median specificity (approx. 80 to approx. 100% in [3] and 93 to 96% in our collective). Of note, an analysis stratified for grading showed that the sensitivities of our single or combined markers with up to 58% by far exceeded those reported for urine cytology (median 12% in grade 1 and median 26% in grade 2 tumors) in our low-grade collective (n = 195) and also in our high-grade collective (n = 53) with up to 72 vs. median 64% in the literature (Table S9, [3]). However, the sensitivities for high-grade detection might be less valid due to the low share of high-grade cases in our collective. Furthermore, the higher sensitivity detected in the high-grade subcollective might be a masked effect of tumor history: only 12 of the 53 high-grade cases had a history of UCa and, as discussed above, people under surveillance mostly have smaller tumors with a lower probability to be detected by molecular markers. This assumption would be in line with our previous observation that DNA methylation levels appear to be independent of grade but are influenced by the history of UCa [12]. 

## 3. Materials and Methods

### 3.1. Urine Specimens

The collective analyzed in the present study was Caucasian and displayed a large overlap with that of our previous study [12] but, for improved analyses, we expanded our previous dataset by newly obtained specimens of male patients. We focused on the male gender for several reasons: the latter would benefit the most from a biomarker-based approach due to the much higher number of men affected by UCa [1] and due to more severe adverse effects caused by cystoscopy in men [2]. Furthermore, we previously showed that our urinary DNA methylation markers are suitable for the identification of UCa in male individuals while we could not presume a robust and reliable use in women. The latter was due to two aspects: First, the incidence of UCa is about four times lower in women than in men [1], entailing a too small number of specimens for a meaningful analysis especially when subgroups were examined [12]. Second, we previously observed a less eminent hypermethylation of biomarker DNA in urine, but not in tissue, from women with UCa when compared to non-UCa specimens [12]. We ascribed that aspect to the higher abundance of non-methylated leukocytes and further non-urothelial cells that suppress DNA methylation in the urinary cell pellets obtained from female UCa patients. [12]. 

The dataset analyzed in the present study fully comprises the male modeling dataset from the previous study [12] and it was complemented by 44 specimens (30 UCa, 9 PCt and 5 UCt samples to a total of 251 UCa patients and 111 controls (Table S1). Histopathological evaluation (staging and grading) was carried out in specimens obtained during transurethral resection of the bladder (TUR-B) by two pathologists based on microscopic evaluation and according to the 2004 WHO guidelines [39]. The control collective consisted of 40 population controls, i.e., individuals without a history of UCa that have been selected from the general population, and 71 urological controls, i.e., patients that underwent (TUR-B) due to the initial suspicion of UCa but where histological examination of specimens did not confirm UCa. Urinary specimen with >500 leukocytes/µL were excluded, as we found that high leukocyte counts would suppress urinary DNA methylation due to a dilution with unmethylated leukocyte DNA [12]. The composition of the analyzed collective regarding staging, grading, localization of UCa and history of UCa and urinary leukocyte counts is given in Table S1.

In contrast to our previous study, we combined both PCt and UCt in a single “no UCa” group because, aside from simplifying analyses, a combined control group is also more representative of the "real-world scenario" where practitioners must decide whether an individual should undergo TUR-B. All urine analyses were approved by the Ethics Committee of the Ruhr-University Bochum (No. 3674-10). The study followed the Declaration of Helsinki and all participants provided written informed consent.

### 3.2. Preparation of DNA from Urine

The isolation of DNA from urine has been described in detail before [12]. In brief, the voided urine was centrifuged and the obtained sediment was washed with PBS and resolved in PBS. DNA was prepared using the QIAmp MinElute Virus Spin Kit (Qiagen, Hilden, Germany) where RNA was digested by incubating the samples with DNase-free RNase (Roche, Mannheim, Germany). DNA was finally purified by the Clean and Concentrator TM-25 Kit (Zymo Research, Corporation, Irvine, CA, USA), eluted in TRIS/EDTA buffer (AppliChem, Darmstadt, Germany) and stored at −20 °C until further analysis.

### 3.3. Quantitative Mass Spectrometry of DNA Methylation

Ten amplicons that were shown to be hypermethylated in UCa cases and whose genomic coordinates and primer sequences have been described previously [12] were analyzed by matrix-assisted laser desorption/ionization–time-of-flight (MALDI-TOF) mass spectrometry (MassARRAY EpiTYPER system, Agena Bioscience GmbH, Hamburg, Germany), enabling the quantitative measurement of CpG methylation at single dinucleotide resolution [40,41]. For this purpose, 250 ng of DNA were bisulfite-converted using the EZ DNA Methylation Gold Kit (Zymo Research, Orange, CA, USA) according to the manufacturer’s protocol. All necessary preparations preceding mass spectrometry (PCR, reverse transcription and RNA cleavage) and the final MALDI-TOF mass spectrometry was performed as previously described [12]. When neighboring CpGs were located on identical mass fragments and could not be resolved at the individual level, a comprehensive DNA methylation value for the whole “CpG unit” was obtained. CpGs with ambiguous mass results, e.g., due to disturbing signals resulting from CpG-free masses, have been excluded from the analysis.

### 3.4. Imputation and Exclusion of CpGs with Missing Values and ROC Curves

We excluded CpGs that contained contained >5% missing values from our analyses and applied our workflow to the remaining 65 CpGs. In these, the few remaining missing values at the CpG level were substituted by imputation. As CpGs from the same amplicon are highly correlated [12], we identified the highest correlated available CpG within the same amplicon. Based on the relationship of the missing and replacing CpG, we then calculated a linear model and used the predicted values from this model to substitute the missing value (Figure S1).

For each of the CpGs a receiver operating characteristic (ROC) analysis was performed using the R package ROCR (version 1.0-11) to assess how well it can distinguish UCa patients from controls. The threshold for each individual CpG was determined at a pre-set specificity of ≥95% and the corresponding sensitivity and accuracy were calculated.

All calculations and graphics were generated using R version 4.0.3 (R Core Team, 2020, Vienna, Austria). Beyond the mentioned R packages at the different steps, the packages openxlsx (version 4.2.3) and rlist (version 0.4.6.1) were used.

### 3.5. Tree-Based Classification Methods

**Figure 1:**
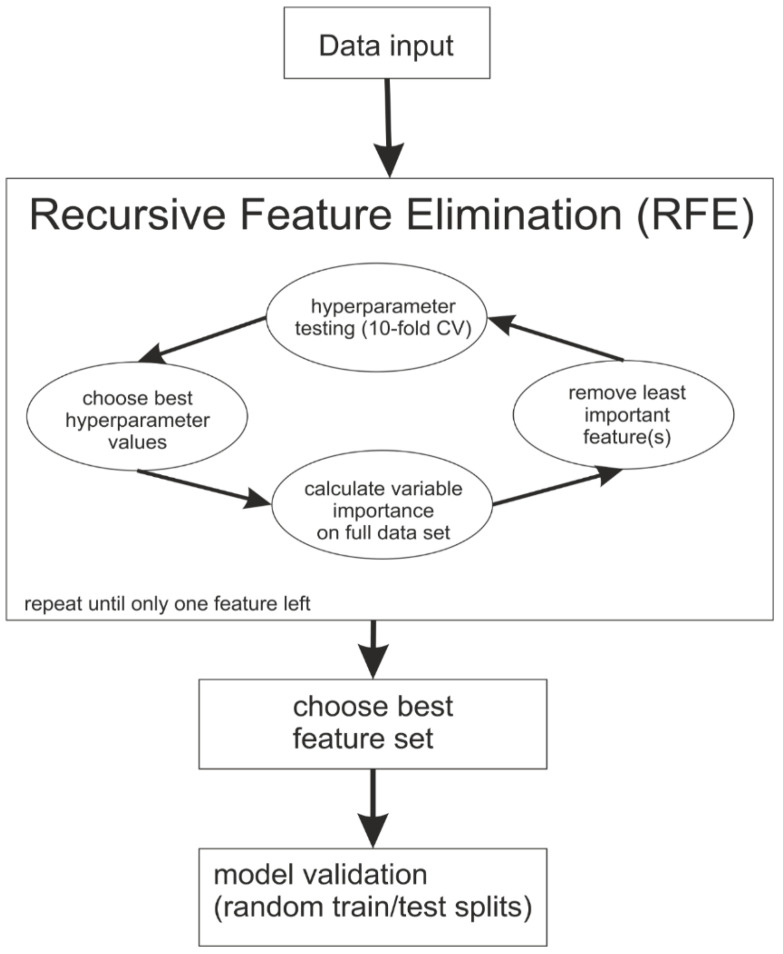
Flow chart describing the modeling process during the applied machine learning approaches and describing the recursive feature elimination.

Both random forest and boosted trees analyses have been used (Figure 1). A recursive feature elimination (RFE) was carried out in which, step by step, the least important features for the separation of UCa and controls were omitted. Different values for the hyperparameters, i.e., settings of the machine learning algorithms, were tested in each iteration to find the best settings for the particular set of CpGs (Table S2, [42,43]). For each possible combination of the considered hyperparameter values, a random forest or gradient-boosted trees model was calculated and evaluated using a 10-fold cross validation with down-sampling (to account for unequal group sizes). Depending on the selection criterion, the hyperparameter values yielding the model with the largest accuracy or specificity were chosen. In a re-training step, the model was calculated again with the optimal hyperparameters employed on the whole dataset. Using this model, the feature importance was calculated for all CpGs. For functionality of hyperparameter tuning and model evaluation, the packages caret (version 6.0-86) and ModelMetrics (version 1.2.2.2) were used.

For random forest (RF), the feature importance is based on the mean decrease in accuracy (MDA). The basic idea is that the accuracy will dramatically decrease if an important feature is randomly permuted [44]. 

For the boosted trees (BT), the feature importance is the total gain in accuracy and is derived from the splits the specific feature is used for divided by the total gain of all features. Therefore, the feature importance is given as a proportion of the sum of all importance values that sum up to 1 over all features.

In both RF and BT, higher feature importance values mean a higher impact on the resulting model. After computing the feature importance, the least important feature was excluded. In the case of multiple least important features that were not used in the BT-derived models, all of them were excluded in that same step. This procedure was repeated until no feature was left. The feature set which led to the highest accuracy (RF_acc_ and BT_acc_) or respective specificity (RF_spec_ and BT_spec_) was selected. In case of a tie, the panel with fewer features was selected. For RF and BT, the R packages randomForest (version 4.6-14) and xgboost (version 1.3.2.1) were used.

### 3.6. Parameter Shrinkage Methods

The least absolute shrinkage and selection operator (LASSO) was applied as an additional method for feature selection as previously described [45]. In brief, the coefficients of some features are shrunk to zero, which can be used for feature selection by selecting features with a non-zero coefficient. The degree of the shrinkage is determined via a hyperparameter lambda, which was chosen as the largest value, such that the cross-validated error is within one standard error of the minimal error of the model. The LASSO analysis was performed using glmnet (version 4.1-1).

### 3.7. Data Validation

To validate the various feature sets which have been identified by the different classification methods, the data were repeatedly split into a training and test dataset 1000 times. Two-thirds of the controls were used for training and one-third was used for testing. To avoid bias for the calculation of the accuracy, the sizes of the training and testing data for the UCa group were based on the same absolute numbers (down-sampling). For each iteration, the accuracy, sensitivity, specificity and area under the ROC curve (AUC) were calculated on the test set. The predicted class probabilities and the true classes for each iteration were combined to a single ROC curve and AUC value for each validation step.

### 3.8. Selection of CpGs for Combinatorial Analyses

As a prerequisite for the identification of UCa patients by way of single CpGs or a panel of CpG sites, the individual threshold at 95% specificity was calculated for all CpGs analyzed (Table S3). In case this threshold was exceeded for the respective singular CpG or a minimum of one CpG in a panel, the individual of interest would be classified as UCa-positive. CpGs concordantly selected in RF, BT and LASSO approaches were considered as candidates to be used in a simple diagnostic decision matrix alone or in combination (Table 1 and Table S8). To select the single marker or marker combination of the highest diagnostic relevance, the sensitivity at 95% specificity was calculated for all CpGs or combinations of CpGs that had been concordantly selected from all statistical approaches (Table 2). The best marker combination for a diagnostic decision matrix was identified according to its diagnostic performance in the overall collective. In addition, sensitivity and specificity were also evaluated in subcollectives stratified for a history of UCa (Table 2).

## 4. Conclusions

Overall, our results, using UCa and DNA methylation as an example, show that the use of multiple bioinformatic methods is highly effective for identifying the most sensitive and specific biomarkers. By using the thresholds determined for the single sites and combining those markers that have been concordantly selected by all bioinformatic approaches, the overall number of potential biomarkers can be effectively reduced to the most reliable ones regarding sensitivity and specificity. In our case, 64 potentially diagnostic CpG sites for UCa could be reduced to two CpG sites, one of each located in *ALOX5* and *TRPS1*, thus allowing for the application of a much cheaper and simpler decision matrix for physicians (Table 3). Like previous findings on UCa-specific hypermethylated CpG sites of other research groups, our results need to be confirmed in a prospective study in urological patients with properly selected inclusion criteria such as, among others, age, gender, microhematuria and/or history of UCa. Ideally, such a study should also consider DNA methylation markers that have been previously reported by other research groups and have been subject to a similarly rigorous selection process. There are few studies that have observed methylation differences among bladder cancer patients of different ethnic affiliations [46,47,48]. Because the collective ethnicity investigated here was Caucasian, the clinical value of our decision matrix needs to be verified in other respective ethnicities before applying it to non-Caucasian individuals, and ethnicity should be considered when planning future cross-sectional and longitudinal validation studies.

## Figures and Tables

**Table 1:** Overview over the results of the applied parameter selection and machine learning approaches in terms of the diagnostic parameters obtained for the individual approaches. Area under the receiver operator curves (AUC) and the respective accuracy and sensitivity are shown. In addition, the sensitivity at a fixed 95% specificity is given. For the statistical and bioinformatical approaches, the number of selected targets and the target names are given. For the machine learning approaches, the number of rounds and the depth/mtry are given.

Applied Approach	Analysis Optimized for.	AUC	Accuracy	Sensi-tivity	Speci-ficity	Sensitivity at ≥95% Specificity	Number of Selected Targets	Rounds	Depth/ Mtry	Selected Targets
**Boosted Trees**	Accuracy	85	78	75	81	53	10	10	4	35_5 and 7 2_6 and 11 78_2.3 and 12.13.14 71_5 14_7.8.9 and 14_1 64_3
	Specificity	84	78	73	83	50	13	6	2	35_5 and 7 78_2.3 and 12.13.14 2_6 and 7 14_1 and 6 74_9 71_8.9.10 and 3.4 and 11 and 5
**Random Forest**	Accuracy	85	79	81	77	51	49	-	3	35_2.3 and 7 and 5 78_2.3 and 12.13.14 and 7.8 and 6 2_6 and 3 and 8 and 2 and 9.10 and 4 and 1 and 11 and 7 71_11 and 8.9.10 and 6.7 and 3.4 and 12 and 5 and 2 14_7.8.9 and 2.3 and 6 and 1 and 10 and 13 and 4.5 and 11.12 and 14 70_3.4 and 6 and 5 and 9.10 and 7 42_10 and 9 and 6.7.8 and 4.5 and 1 and 12 74_8 and 10.11.12 and 6 and 3.4 and 9 64_3
	Specificity	85	79	81	77	52	43	-	2	78_2.3 and 12.13.14 and 6 and 7.8 35_ 5 and 2.3 and 7 2_ 8 and 6 and 3 and 2 and 1 and 7 and 11 and 4 and 9.10 71_11 and 3.4 and 8.9.10 and 6.7 and 5 and 12 and 14_1 and 6 and 2.3 and 11.12 and 10 and 14 and 13 and 7.8.9 74_8 and 6 and 3.4 70_3.4 and 7 and 6 42_2.3 and 9 and 10 and 4.5 and 1 and 12
**LASSO**		85	79	71	86	52	4	-	-	02_6 35_7 42_9 78_2.3
**Singular CpGs with** **>50% sensitivity at** **≥95% specificity**		max. 86 for 78_2.3				max. 58 for 2_6	21	-	-	02_6 and 8 and 3 and 4 and 2 and 9.10 and 11 and 7 78_6 14_11.12 and 10 and 7.8.9 and 2.3 and 13 70_6 74_6 22_2 and 3 35_7 42_1 and 10

**Table 2:** Sensitivities and specificities for single CpGs/CpG-units or panels consisting of up to three candidate sites selected by RF, BT and LASSO in the overall dataset and stratified for UCa history. Sites or, respectively, panels were ranked for the best overall specificity, and, within the latter, sensitivity. Sites or combinations with less than 95% overall specificity were not considered further (gray text).

	Overall		No UCa History		UCa History	
Single CpG/CpG-Unit or Panel	Sensitivity	Specificity	Sensitivity	Specificity	Sensitivity	Specificity
**02_CpG_6**	58	96	70	98	40	94
**02_CpG_6; 35_CpG_7**	61	95	72	98	43	90
**35_CpG_7**	50	95	59	100	37	90
**78_CpG_2.3**	43	95	54	98	28	92
**02_CpG_6; 35_CpG_7; 78_CpG_2.3**	61	93	73	98	43	86
**35_CpG_7; 78_CpG_2.3**	54	93	65	98	37	86

**Table 3:** DNA methylation thresholds of the diagnostic targets *ALOX 5* and *TRPS1*. In case the threshold is exceeded for one of the targets, the patient should be stratified for cystoscopy.

Target	Threshold DNA-Methylation at 95% Specificity
**02_CpG6 (*ALOX5*)**	0.435
**35_CpG7 (*TRPS1*)**	0.465

## Data Availability

Data beyond those presented here are available in the Supplementary Material.

## Supplementary Materials

The supporting information can be downloaded at: https://www.mdpi.com/article/10.3390/ijms25020738/s1.
