# Supplementary material for: Use of Multiple Machine Learning Approaches for Selecting Urothelial Cancer-Specific DNA Methylation Biomarkers in Urine
Source: Int J Mol Sci. 2024 Jan 6;25(2):738. doi: 10.3390/ijms25020738 (PMC10815677; doi:10.3390/ijms25020738)
Supplement: Supplementary file 1 [file ijms-25-00738-s001.zip › ijms-2688446-supplementary.pdf]

Use of multiple machine learning approaches for selecting Urothelial cancer specific DNA-methylation biomarkers in urine – Supplementary Material

SUPPLEMENTARY TABLES

	UCa	PCt	UCt	no Uca
	male	male	male	male
n	251	40	71	111
Age in years median	72	70.5	69	70
Age in years range	(35-92)	(48-87)	(41-86)	(41-87)
Staging UCa				
Ta	128			
T1	87			
T2	27			
Tis	16			
N/A	3			
Grading UCa (n)				
Low grade	195			
High grade	53			
N/A	3			
Localization UCa				
Bladder	143			
Ureter	6			
Urethra	2			
History of UCa (n)				
No	151	39	21	60
Yes	100	1	50	51
Leukocytes in urine (n)				
0	161	33	42	75
25 Leu/µL	67	4	22	26
100 Leu/µL	46	3	14	17

Table S1: Patient and urine sample characteristics. The study population was stratified for history of urothelial carcinoma, tumor classification and urinary leukocyte counts. UCa=urothelial carcinoma; UCt=urological controls; PCt=population controls; Cis=carcinoma in situ.

Description	Machine learning method	min	max	stepsize
**maximum depth of a tree**	boosted trees	2	10	2
**maximal number of boosting iterations**	boosted trees	1	20	1
**number of candidate features considered in each branching within the tree**	random forest	1	floor(sqrt(p))	1

Table S2. Values considered for the hyperparameters. p = number of remaining features in this iteration; floor = rounding to the greatest integer less than or equal to the original number; sqrt = squareroot

Amplicon and CpG	AUC	threshold at minimum 95% specificity	sensitivity at the threshold with minimum 95% specificity	sensitivity at the threshold with minimum 95% specificity
02_CpG_1	0,80991	0,335	0,40239	0,95495
02_CpG_2	0,83299	0,445	0,54183	0,95495
02_CpG_3	0,83769	0,375	0,55378	0,95495
02_CpG_4	0,83028	0,445	0,54980	0,95495
02_CpG_6	0,84582	0,435	0,57769	0,96396
02_CpG_7	0,82775	0,295	0,50199	0,95495
02_CpG_8	0,83500	0,415	0,56972	0,95495
02_CpG_9.10	0,83432	0,345	0,53386	0,95495
02_CpG_11	0,80864	0,535	0,53386	0,95495
14_CpG_1	0,77709	0,485	0,47809	0,95495
14_CpG_2.3	0,79114	0,475	0,50996	0,95495
14_CpG_4.5	0,79685	0,435	0,48207	0,95495
14_CpG_6	0,79572	0,415	0,45418	0,96396
14_CpG_7.8.9	0,79857	0,395	0,51793	0,95495
14_CpG_10	0,80640	0,475	0,52191	0,95495
14_CpG_11.12	0,79965	0,445	0,53386	0,95495
14_CpG_13	0,79419	0,385	0,50199	0,95495
14_CpG_14	0,77869	0,425	0,45020	0,95495
22_CpG_2	0,79651	0,345	0,50598	0,95495
22_CpG_3	0,79602	0,375	0,50199	0,95495
22_CpG_4	0,77876	0,415	0,49402	0,95495
22_CpG_5	0,78244	0,475	0,47410	0,95495
22_CpG_8	0,78484	0,465	0,46614	0,95495
22_CpG_9	0,78303	0,47	0,47410	0,96396
35_CpG_2.3	0,85388	0,405	0,49801	0,96396
35_CpG_5	0,85018	0,375	0,49801	0,95495
35_CpG_7	0,84767	0,465	0,50199	0,95495
42_CpG_1	0,82682	0,445	0,50199	0,96396
42_CpG_2.3	0,81908	0,355	0,46215	0,95495
42_CpG_4.5	0,81691	0,365	0,43825	0,96396
42_CpG_6.7.8	0,81602	0,405	0,46215	0,95495
42_CpG_9	0,83123	0,435	0,49402	0,95495
42_CpG_10	0,81831	0,435	0,50199	0,95495
42_CpG_12	0,82434	0,475	0,49801	0,96396
64_CpG_1	0,79470	0,455	0,41833	0,96396
64_CpG_2	0,77910	0,465	0,41833	0,96396
64_CpG_3	0,80483	0,515	0,43825	0,95495
64_CpG_4	0,78549	0,465	0,41833	0,95495
64_CpG_5	0,78258	0,405	0,41434	0,95495
64_CpG_7.8	0,78482	0,745	0,39841	0,95495
70_CpG_3.4	0,79236	0,475	0,49004	0,95495
70_CpG_5	0,78692	0,505	0,46614	0,95495
70_CpG_6	0,80013	0,515	0,52590	0,95495
70_CpG_7	0,72858	0,545	0,38247	0,95495
70_CpG_9.10	0,80912	0,395	0,44223	0,95495
70_CpG_14	0,78310	0,395	0,41036	0,95495
71_CpG_1	0,75184	0,395	0,37052	0,96396
71_CpG_2	0,75182	0,435	0,33466	0,96396
71_CpG_3.4	0,75267	0,485	0,40637	0,95495
71_CpG_5	0,71803	0,465	0,33865	0,95495
71_CpG_6.7	0,76623	0,555	0,37052	0,95495
71_CpG_8.9.10	0,78712	0,535	0,49402	0,95495
71_CpG_11	0,79498	0,495	0,48606	0,96396
71_CpG_12	0,74611	0,525	0,37450	0,95495
74_CpG_3.4	0,82431	0,435	0,48207	0,95495
74_CpG_5	0,77743	0,315	0,34661	0,95495
74_CpG_6	0,81040	0,325	0,52191	0,95495
74_CpG_7	0,76338	0,365	0,33865	0,95495
74_CpG_8	0,80299	0,245	0,45817	0,95495
74_CpG_9	0,78863	0,265	0,43426	0,95495
74_CpG_10.11.12	0,82414	0,305	0,42231	0,95495
78_CpG_2.3	0,86009	0,555	0,43426	0,95495
78_CpG_6	0,83559	0,355	0,56972	0,95495
78_CpG_7.8	0,83823	0,395	0,49402	0,95495
78_CpG_12.13.14	0,83540	0,275	0,47410	0,95495

Table S3: AUCs as well as sensitivities and specificities referring to the threshold at minimum 95% specificity for the individual CpGs.

Variable	Gain
**35_CpG_2,3**	10,66861304
**35_CpG_7**	10,61954113
**78_CpG_2,3**	9,731272295
**35_CpG_5**	9,320422225
**02_CpG_6**	8,912009401
**71_CpG_11**	7,830720814
**02_CpG_3**	7,721395808
**71_CpG_8,9,10**	7,669981896
**14_CpG_7,8,9**	7,6578913
**70_CpG_3,4**	7,657756872
**02_CpG_8**	7,269367192
**14_CpG_2,3**	7,211795598
**02_CpG_2**	6,940414613
**78_CpG_12,13,14**	6,939075511
**42_CpG_10**	6,550684244
**14_CpG_6**	6,530556086
**02_CpG_9,10**	6,501367669
**14_CpG_1**	6,460030774
**14_CpG_10**	6,382847978
**71_CpG_6,7**	6,36937089
**14_CpG_13**	6,330781662
**71_CpG_3,4**	6,323396691
**42_CpG_9**	6,277232649
**71_CpG_12**	6,270517276
**71_CpG_5**	6,187823415
**02_CpG_4**	6,061638874
**74_CpG_8**	6,040305118
**70_CpG_6**	5,814425711
**42_CpG_6,7,8**	5,739391334
**42_CpG_4,5**	5,625542884
**78_CpG_7,8**	5,580667898
**70_CpG_5**	5,538830509
**14_CpG_4,5**	5,379087331
**70_CpG_9,10**	5,303323403
**02_CpG_1**	5,239501335
**42_CpG_1**	5,075229702
**42_CpG_12**	5,051759574
**02_CpG_11**	5,043531967
**74_CpG_10,11,12**	5,01752708
**78_CpG_6**	4,917015972
**14_CpG_11,12**	4,884710782
**02_CpG_7**	4,845839163
**14_CpG_14**	4,83514427
**71_CpG_2**	4,717796786
**70_CpG_7**	4,468297992
**74_CpG_6**	4,363758733
**64_CpG_3**	4,098294378
**74_CpG_3,4**	3,970109515
**74_CpG_9**	2,017618288

Table S4: Variables and gains for the random forest accuracy analysis presented in the order of descending gains

Variable	Gain
**78_CpG_2,3**	9,625572037
**35_CpG_5**	8,834559128
**35_CpG_2,3**	8,81596353
**02_CpG_8**	8,374873036
**71_CpG_11**	7,955909066
**35_CpG_7**	7,86994805
**14_CpG_1**	7,691645996
**02_CpG_6**	7,685204454
**14_CpG_6**	7,561897303
**02_CpG_3**	7,43457735
**71_CpG_3,4**	7,256460684
**74_CpG_8**	7,218894677
**78_CpG_12,13,14**	7,141516157
**70_CpG_3,4**	7,022778801
**71_CpG_8,9,10**	6,966229018
**14_CpG_2,3**	6,880071432
**14_CpG_11,12**	6,854446553
**02_CpG_2**	6,319375511
**70_CpG_7**	6,265230679
**02_CpG_1**	6,263411987
**70_CpG_6**	6,159510296
**02_CpG_7**	6,158493869
**71_CpG_6,7**	6,036166203
**02_CpG_11**	5,994626294
**42_CpG_2,3**	5,96984985
**78_CpG_6**	5,912002755
**42_CpG_9**	5,856165543
**74_CpG_6**	5,813733479
**78_CpG_7,8**	5,689455605
**71_CpG_5**	5,589377261
**71_CpG_12**	5,569047066
**02_CpG_4**	5,524074837
**42_CpG_10**	5,501817027
**42_CpG_4,5**	5,476061516
**14_CpG_10**	5,468554455
**14_CpG_14**	5,394757874
**42_CpG_1**	5,325541878
**02_CpG_9,10**	5,243466684
**14_CpG_13**	4,827112228
**70_CpG_9,10**	4,749427402
**14_CpG_7,8,9**	4,420146447
**42_CpG_12**	4,418998182
**74_CpG_3,4**	4,214448991

Table S5: Variables and gains for the random forest specificity analysis presented in the order of descending gains

Variable	Gain
35_CpG_5	0,291474608
02_CpG_6	0,126829194
35_CpG_7	0,120671732
78_CpG_2,3	0,11326474
71_CpG_5	0,079259683
02_CpG_11	0,073051075
14_CpG_7,8,9	0,060040128
14_CpG_1	0,058205416
64_CpG_3	0,040574752
78_CpG_12,13,14	0,036628671

Table S6: Variables and gains for the boosted trees accuracy analysis presented in the order of descending gains

Variable	Gain
35_CpG_5	0,29447613
35_CpG_7	0,237574358
78_CpG_2,3	0,194684897
02_CpG_6	0,086141723
78_CpG_12,13,14	0,044190993
14_CpG_1	0,025731423
74_CpG_9	0,023502392
14_CpG_6	0,023280829
71_CpG_8,9,10	0,022261492
71_CpG_3,4	0,019780189
02_CpG_7	0,016272253
71_CpG_11	0,006312997
71_CpG_5	0,005790323

Table S7: Variables and gains for the boosted trees specificity analysis presented in the order of descending gains

	Boosted trees		Random Forest		LASSO
	accuracy	specificity	accuracy	specificity	
2_CpG_6	X	X	X	X	X
35_CpG_7	X	X	X	X	X
78_CpG_2.3	X	X	X	X	X
14_CpG_1	X	X	X	X	
71_CpG_5	X	X	X	X	
35_CpG_5	X	X	X	X	
78_CpG_12.13.14	X	X	X	X	
42_CpG_9			X	X	X
2_CpG_11	X		X	X	
14_CpG_7.8.9	X		X	X	
14_CpG_6		X	X	X	
71_CpG_11		X	X	X	
71_CpG_3.4		X	X	X	
71_CpG_8.9.10		X	X	X	
2_CpG_7		X	X	X	
2_CpG_1			X	X	
2_CpG_2			X	X	
2_CpG_3			X	X	
2_CpG_4			X	X	
2_CpG_8			X	X	
2_CpG_9.10			X	X	
14_CpG_10			X	X	
14_CpG_11.12			X	X	
14_CpG_13			X	X	
14_CpG_14			X	X	
14_CpG_2.3			X	X	
35_CpG_2.3			X	X	
42_CpG_1			X	X	
42_CpG_10			X	X	
42_CpG_12			X	X	
42_CpG_4.5			X	X	
64_CpG_3	X		X		
64_CpG_4	X		X		
70_CpG_3.4			X	X	
70_CpG_6			X	X	
70_CpG_7			X	X	
70_CpG_9.10			X	X	
71_CpG_12			X	X	
71_CpG_6.7			X	X	
74_CpG_3.4			X	X	
74_CpG_6			X	X	
74_CpG_8			X	X	
74_CpG_9		X	X		
78_CpG_6			X	X	
78_CpG_7.8			X	X	
14_CpG_4.5			X		
42_CpG_2.3				X	
42_CpG_6.7.8			X		
70_CpG_5			X		
71_CpG_2			X		
74_CpG_10.11.12			X		

Table S8: Selection of investigated sites with < 5% missings by the different indicated CpGs. Sites are ordered according to the number of approaches they were selected by, in descending order. Sites that were not chosen by any approach are not listed.

	senstivity	
Single CpG / CpG-unit or panel	low grade	high grade

**02_CpG_6**	55	70
**02_CpG_6;35_CpG_7**	**58**	**72**
**35_CpG_7**	51	49
**78_CpG_2.3**	42	51
**02_CpG_6;35_CpG_7;78_CpG_2.3**	58	73
**35_CpG_7;78_CpG_2.3**	52	70

Table S9: Sensitivities for single CpGs / CpG-units or panels consisting of up to 3 candidate sites selected by RF, BT and LASSO in the UCa subsets of our dataset stratified for grading (n=195 low grade and 53 high grade cases)

SUPPLEMENTARY FIGURES

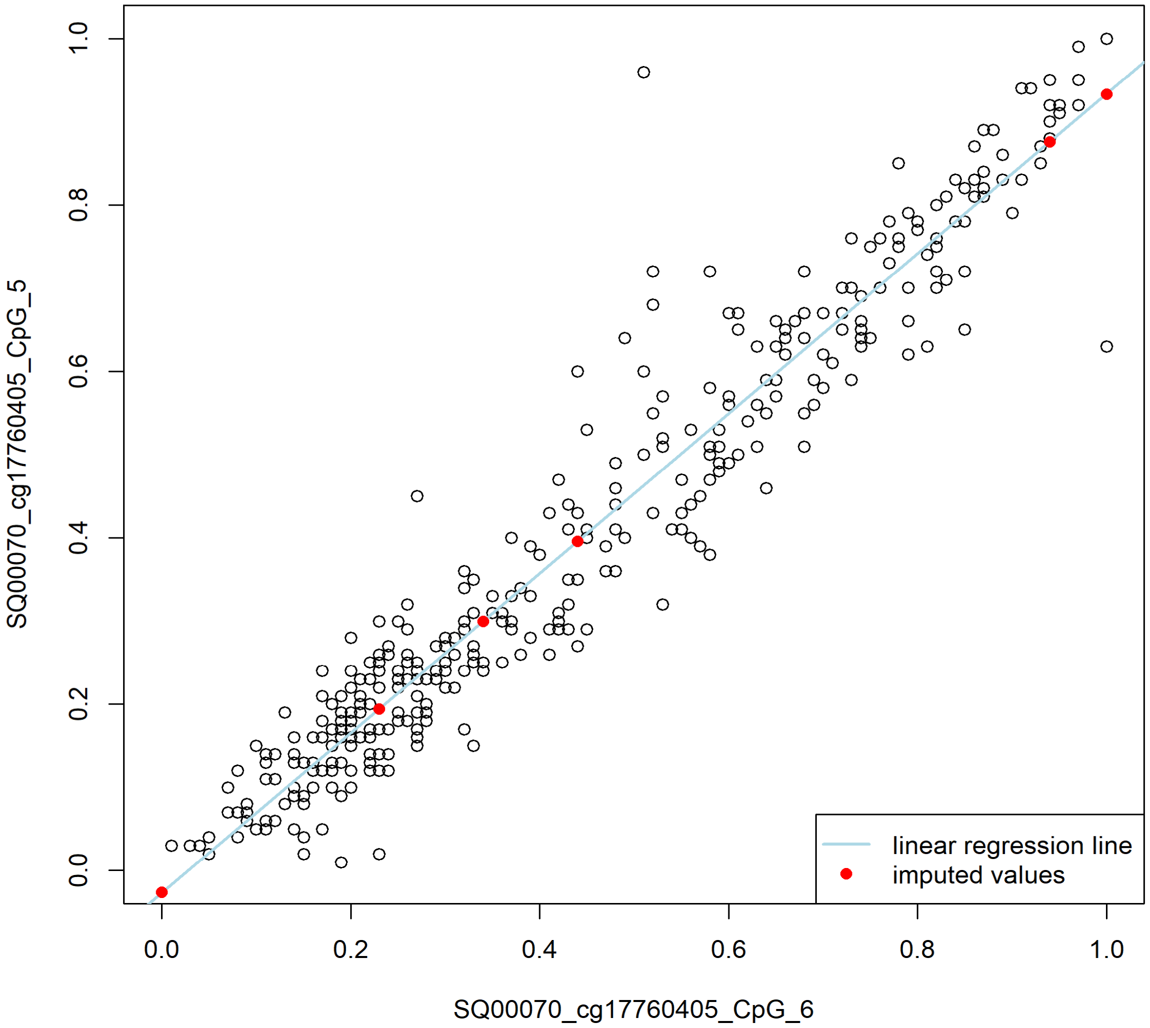

Figure S1: Scatterplot of two exemplarily chosen CpGs to illustrate the imputation method. CpG 70_5 (y-axis) has some missing values. Within the same Amplicon, the CpG with the highest correlation is searched, which is CpG 70_6 (x-axis).

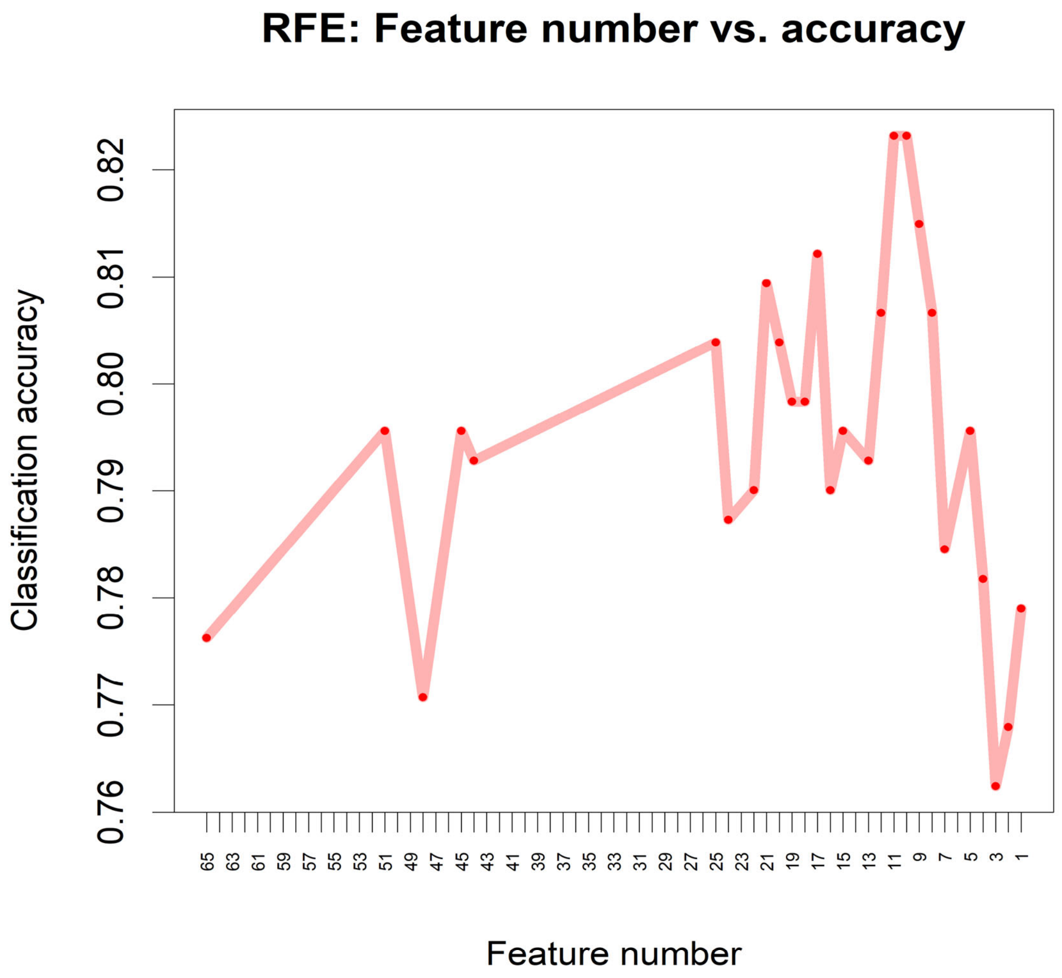

Figure S2. Plot of the achieved accuracy (y-axis) against the number of features in the respective biomarker panel (x-axis) in the boosted trees approach.

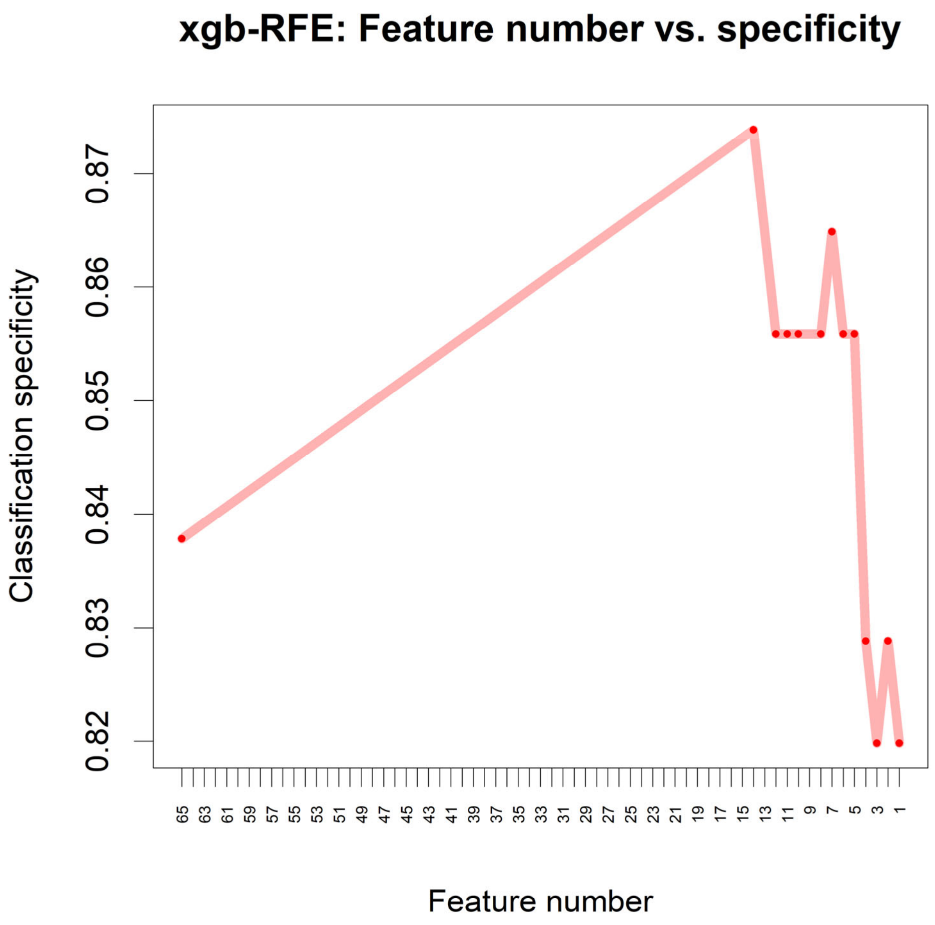

Figure S3. Plot of the achieved specificity (y-axis) against the number of features in the respective biomarker panel (x-axis) in the boosted trees approach.

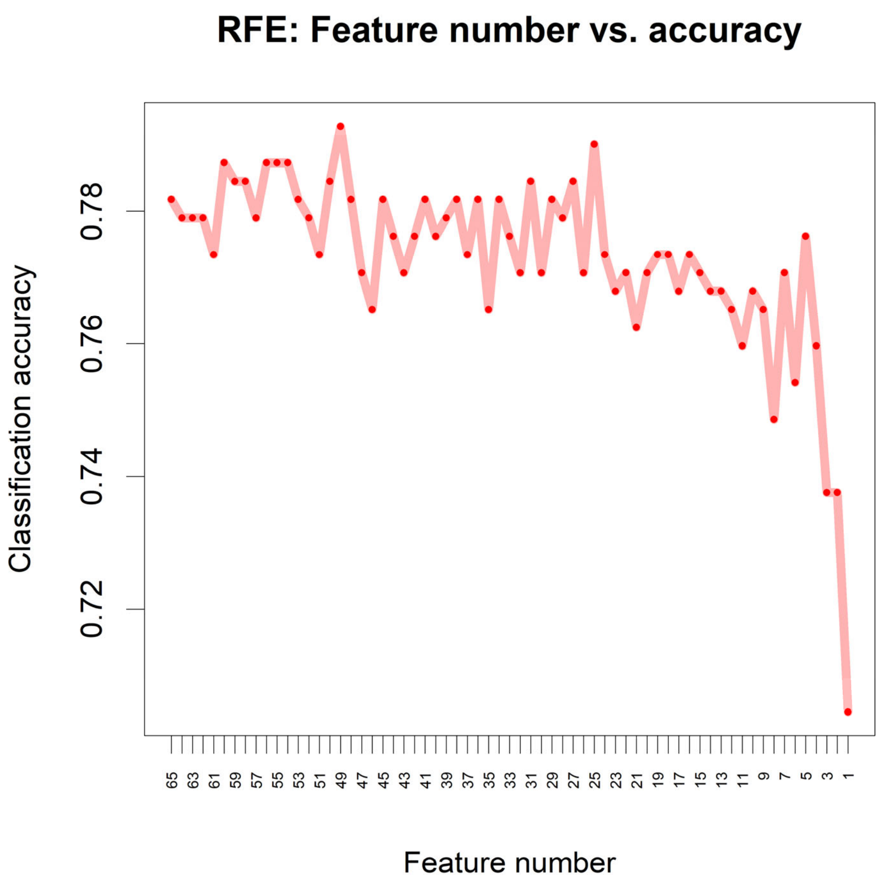

Figure S4. Plot of the achieved accuracy (y-axis) against the number of features in the respective biomarker panel (x-axis) in the random forest approach

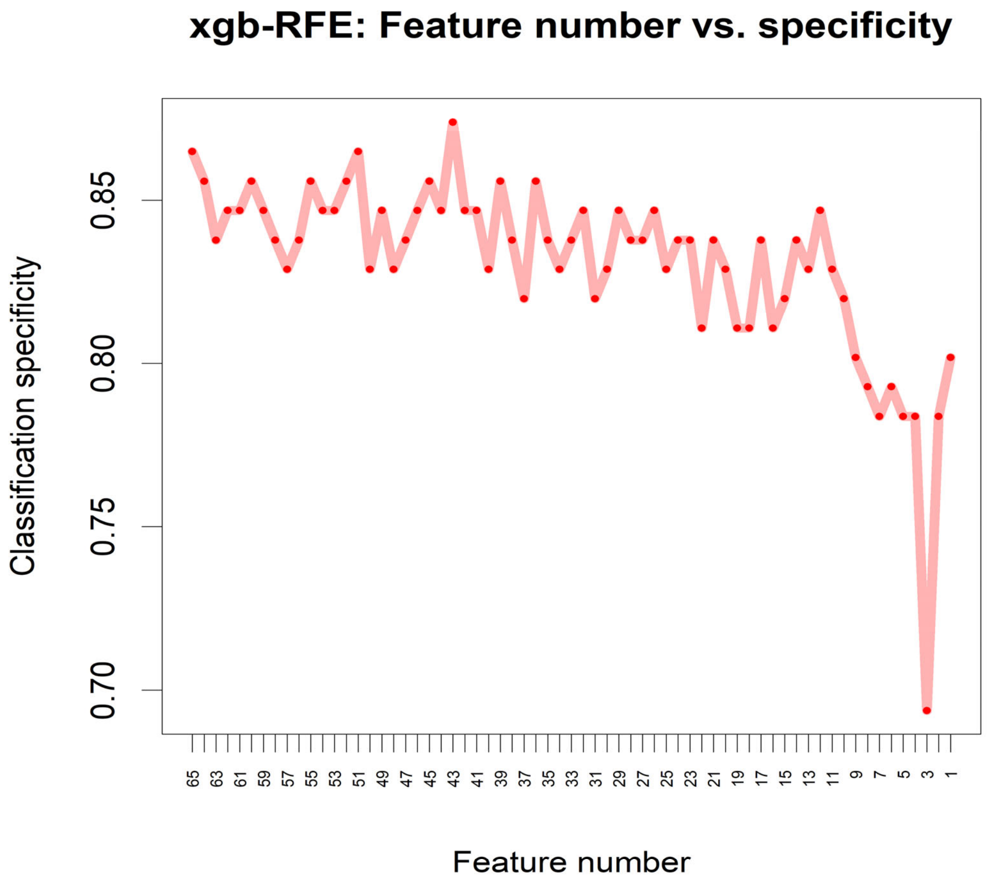

Figure S5. Plot of the achieved specificity (y-axis) against the number of features in the respective biomarker panel (x-axis) in the random forest approach.
